# Sit-to-walk strategy classification in healthy adults using hip and knee joint angles at gait initiation

**DOI:** 10.1038/s41598-023-43148-0

**Published:** 2023-10-03

**Authors:** Chamalka Kenneth Perera, Alpha Agape Gopalai, Darwin Gouwanda, Siti Anom Ahmad, Mazatulfazura Sf Binti Salim

**Affiliations:** 1 School of Engineering, Monash University, Subang Jaya, Selangor Malaysia; 2 Malaysian Research Institute on Ageing, Universiti Putra Malaysia, Serdang, Selangor Malaysia

**Keywords:** Biomedical engineering, Musculoskeletal system, Quality of life

## Abstract

Forward continuation, balance, and sit-to-stand-and-walk (STSW) are three common movement strategies during sit-to-walk (STW) executions. Literature identifies these strategies through biomechanical parameters using gold standard laboratory equipment, which is expensive, bulky, and requires significant post-processing. STW strategy becomes apparent at gait-initiation (GI) and the hip/knee are primary contributors in STW, therefore, this study proposes to use the hip/knee joint angles at GI as an alternate method of strategy classification. To achieve this, K-means clustering was implemented using three clusters corresponding to the three STW strategies; and two feature sets corresponding to the hip/knee angles (derived from motion capture data); from an open access online database (age: 21–80 years; n = 10). The results identified forward continuation with the lowest hip/knee extension, followed by balance and then STSW, at GI. Using this classification, strategy biomechanics were investigated by deriving the established biomechanical quantities from literature. The biomechanical parameters that significantly varied between strategies (*P* < 0.05) were time, horizontal centre of mass (COM) momentum, braking impulse, centre of pressure (COP) range and velocities, COP–COM separation, hip/knee torque and movement fluency. This alternate method of strategy classification forms a generalized framework for describing STW executions and is consistent with literature, thus validating the joint angle classification method.

## Introduction

Sit-to-walk (STW) is a critical weight-bearing activity of daily living (ADL), with adults performing this task approximately sixty times daily^1^. STW takes place when an individual transitions from a seated position to walking, via standing. This common motion, although seemingly basic, plays a major role in ensuring stability during time critical ADLs, for example, when moving from a seated position to answer a telephone or a doorbell. However, literature reports that the ability to execute STW deteriorates with age^2–4^. Losing the ability to perform STW safely not only increases fall risk but also results in physical, psychological, and emotional degradation^5,6^. Therefore, investigating and understanding the biomechanics, characteristics and executions of STW, is a vital first step in ensuring mobility, independent living and a high quality of life, for adults and movement-impaired individuals^2,4^.

At present, there is numerous literature investigating sit-to-stand (STS). However, STS is merely a subset of STW, because it is normal to assume that an individual will ambulate after standing. Hence, in STW the end goal is walking, which is more common in daily life, making it a better representation of ADLs^4^. Thus, STW is defined as a fluid merging of STS and gait, at the point of gait-initiation (GI), where GI is denoted as the heel-off of the swing foot^2^.

**Figure 1:**
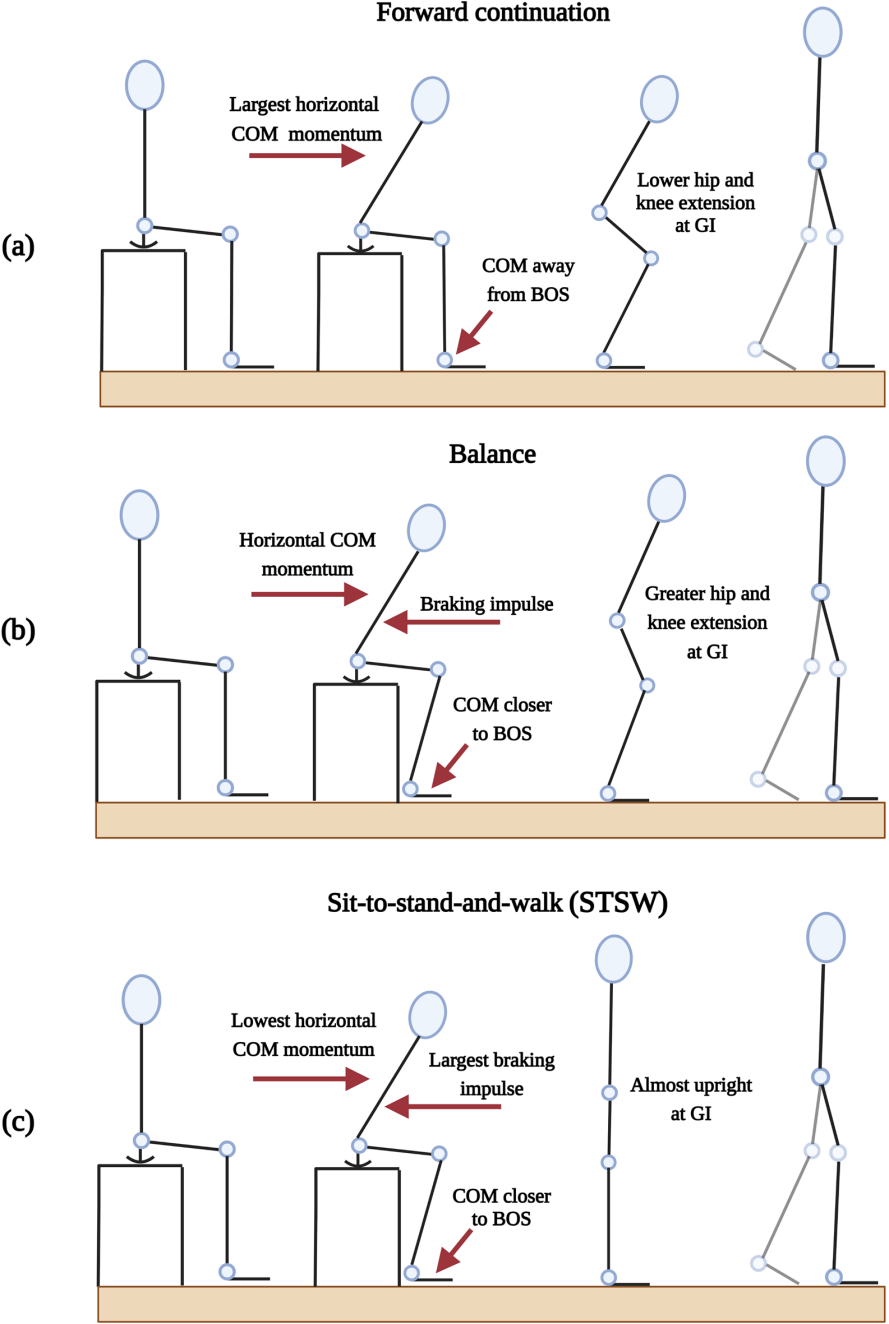
The three STW movement strategies: (**a**) forward continuation (**b**) balance^7,9^ and (**c**) STSW^2^. The levels of horizontal centre of mass (COM) momentum, braking impulse, and the degree of hip and knee extension at gait-initiation (GI) are shown. Additionally, the foot position is given, which affects how close the COM is to the body’s base of support (BOS).

**Figure 2:**
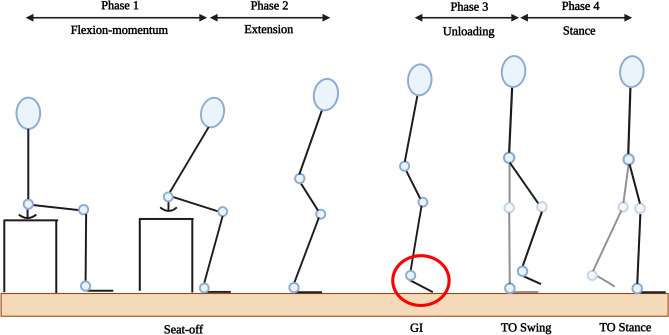
The four transitionary phases of the STW cycle, adapted from Buckley et al.,^2^. This includes flexion-momentum, extension, unloading and stance. Seat-off occurs at the end of flexion-momentum while gait-initiation (GI) occurs at the end of the extension phase and is denoted by the heel-off of the swing foot, as shown circled in red. This is followed by a toe-off (TO) of the swing foot at the end of unloading and TO of the stance foot at the end of the stance phase.

Literature reports of multiple studies that established similar biomechanical variations in STW executions in their subject populations; and how these variations were generalised into different STW movement strategies, although with different nomenclature^2,7–10^. These identified strategies can be generally divided into three groups (Fig. 1): (a) Forward continuation, (b) Balance and (c) Sit-to-stand-and-walk (STSW). Considering this, Magnan et al.,^7^ researched on the anteroposterior (AP) ground reaction forces (GRFs), centre of mass (COM) momentum and displacement, with centre of pressure (COP) trajectories, to differentiate between forward continuation and balance strategies in healthy adults. Similarly, Rousanoglou et al.,^9^ investigated movement speed and duration (fast and preferred speeds), COM velocity and displacement, COP trajectories and the temporal patterns of the STW transition phases (Fig. 2), to also distinguish between such strategies. Likewise, Bestaven et al.,^8^ and Buckley et al.,^2^ considered the total COP trajectory, COM momentum, COP–COM separation and step length/velocity, in relation to ageing to propose an alternate STW strategy commonly seen in older adults (STSW). More recently, Chandler et al.,^11^ and Kerr et al.,^12^ studied the variation in movement fluency during STW, while Jones et al.,^10^ sought to find consistent biomechanical parameters between different STW strategies. From these studies, literature establishes that STW strategies can be explained by a series of biomechanical parameters, which forms a generalized description of the STW executions, despite each subject showing biomechanical variations due to individual execution styles and preferences.

As illustrated in Fig. 1, in forward continuation, a large horizontal COM (hCOM) momentum is generated, to propel the body forwards and upwards, while GI occurs earlier than in the other strategies (closer to seat-off). The feet or base of support (BOS) can be further away from the body (COM), as the generated momentum will carry the individual forwards. In balance, a braking impulse (posterior GRF) occurs to reduce the hCOM momentum generated and maintain quasi-static and postural stability, while rising. GI is delayed and the BOS is closer to the COM, compared to forward continuation^7,9^. While in STSW, a significant braking impulse occurs, with the BOS closest to the COM. This allows the individual to reach an almost upright position before a delayed GI (compared to forward continuation or balance)^2,10^. With this, Dehail et al.,^13^ showed that the quadriceps and hamstrings are the primary muscles involved in STW as they allow for hip and knee extension when rising, while modulating the braking impulse. Therefore, the hip and knee are the primary contributors in STW.

The above literature highlighted the different executions of STW with their strategy-wise biomechanics, based on the investigated biomechanical parameters. To reliably distinguish between the STW strategies, all biomechanical parameters should be considered as literature lacked agreement on the parameters of interest. Additionally, to derive these biomechanical parameters, gold standard laboratory equipment like motion capture (Mocap) systems or force plates are required. Using such equipment is expensive, bulky, computationally heavy and requires significant post-processing^14,15^. Therefore, an alternate method of STW strategy classification using a single, standalone parameter, measurable through wearable sensors, is significant. This would enable strategy identification to be performed outside the laboratory and used in real-time applications.

By considering STW motion biomechanics, strategy executions (Fig. 1) and the definition of STW^16^; the study observed that the strategy first becomes apparent only at GI. This is because, before GI an individual begins rising symmetrically and the strategy is not yet distinguishable; however, after GI, as the swing foot moves forward, the chosen strategy is visible. At GI, the complete hCOM momentum and braking impulse generated are observable, along with the COM relative to the BOS, which determines the chosen strategy. As such, this study hypothesizes that the hip and knee angles at GI can serve as a distinguishing factor in classifying the STW strategy. Lower limb joint angles for strategy classification are beneficial as they are standalone biomechanical parameters and can be easily measured using wearable sensors, in contrast to the Mocap derived biomechanical parameters from literature. Through STW strategy classification, a generalized framework for STW execution biomechanics and characteristics can be described. This would inform the design of interventions by providing reference biomechanical trajectories of healthy adults (for example joint torque) that can be tracked to aid impaired motion^17,18^. This in turn promotes independent living, easier access to ADLs and a better quality of life^19^. In this study, clustering was proposed to classify the strategies into forward continuation, balance and STSW groups, based on the degree of hip/knee extension at GI. Furthermore, the varying STW strategy execution biomechanics and characteristics were investigated, to understand the strategies and for validation with literature.

## Methods

### Experiment details

Data from an open access database by Liang et al.,^20^ performed in the Rehabilitation Research Institute of Singapore, was considered in this study. The data collection was approved by the Nanyang Technological University Institutional Review Board (IRB-2018-04-014), and all subjects provided informed consent before commencement, in accordance with the Declaration of Helsinki. Raw Mocap and force plate data were provided in C3D format, from the NTU Dataverse database^20^. The dataset consisted of ten healthy subjects of Asian ethnicity (weight: 60.6 ± 11.3 kg, height: 166.5 ± 10.9 cm) with a wide age group ranging from 21 to 80 years, inclusive of all three STW strategies. Subjects performed the timed-up-and-go (TUG) test, as detailed in Chen & Chou^21^, with three repetitions, from which STW was obtained. During the TUG test, subjects were asked to stand from a seated position, walk forward for 3 m, turn around, walk back, and sit down. Mocap data was obtained through a Qualisys (Sweden) Mocap system, sampling at 200 Hz, while GRF and COP data were obtained using two Kistler (Switzerland) force plates, sampling at 2000 Hz.

### Data processing

This study processed and analyzed the raw Mocap and GRF data^20^. First, the raw data was filtered to minimize noise, motion artifacts and for smoothing—using a zero-lag, second order, Butterworth lowpass filter with a cut-off frequency of 5 Hz and 20 Hz for Mocap and force plate data, respectively^22,23^. The filter cut-off frequencies were selected by performing a Fast Fourier Transform and observing the 99% occupied signal bandwidth. Additionally, the force plate cut-off frequency was selected to preserve GRF events during GI and to identify its occurrence.

Following this, OpenSim 4.2^24,25^ was used for biomechanical analysis (see Supplementary Information), with STW being modelled using the Gait2392 Musculoskeletal Model^26^. This model was chosen as only the lower limbs were studied, while still accounting for upper body weight. Scaling was performed to match the subject’s anthropometry to the model, followed by inverse kinematics and inverse dynamics, to compute hip and knee joint angles and torques, respectively^27^. Additionally, COM trajectories and velocities were derived through BodyKinematics analysis, while COP and GRFs were obtained directly from the force plates. Subsequently, these biomechanical parameters were analysed using MATLAB (Mathworks Inc.).

### Data analysis

#### Joint angle clustering

As STW is defined as a fluid merging of STS and gait at the point of GI^16^, the three STW strategies become apparent only at GI. After GI, gait begins as the swing foot moves forward at ‘toe-off (TO) Swing’ (Fig. 2), with different execution mechanics. Based on the STW strategy executed, the hip and knee joint angles (which are the primary contributors of STW), vary at GI (Fig. 1), and thus can be used to identify the strategy.

K-means clustering was chosen for STW strategy classification as three distinctly identifiable strategies are known to exist, from literature. It is a fast and established algorithm that can naturally identify these selected groups, within a numerical dataset with similar characteristics^28^. As STW executions are grouped into three strategies, K-mean clustering was performed using three clusters and two data/feature sets—the hip and knee joint angles at GI^2,7^. However, the number of STW trials per strategy were not equal, with eleven for forward continuation, twelve for balance and seven for STSW. Therefore, synthetic minority oversampling technique (SMOTE) was applied, to equalise sample length for each strategy. In total, twelve trials were considered per strategy with joint angles from both left and right legs, resulting in 24 data points per strategy cluster. The cluster range was visualized using a circle, centred at the cluster centroid and radius equal to the Euclidean distance of the furthest point.

Considering this, the strategies were identified based on each cluster centroid’s degree of hip/knee extension at GI. The forward continuation cluster had the lowest hip/knee extension (largest joint angle magnitude) followed by balance (moderate hip/knee extension) and finally STSW—which had the greatest hip/knee extension (lowest joint angle magnitude), as subjects were almost upright at GI. With this, silhouette analysis (describing cluster cohesion and separation) was performed, and produced values greater than zero, showing that no data points were wrongly assigned to a cluster^29^. Fuzzy C-means clustering was also performed using the hip and knee joint angles at GI and produced identical results to that from K-means clustering, thus validating the K-means clustering results.

#### Biomechanical parameters

**Figure 3:**
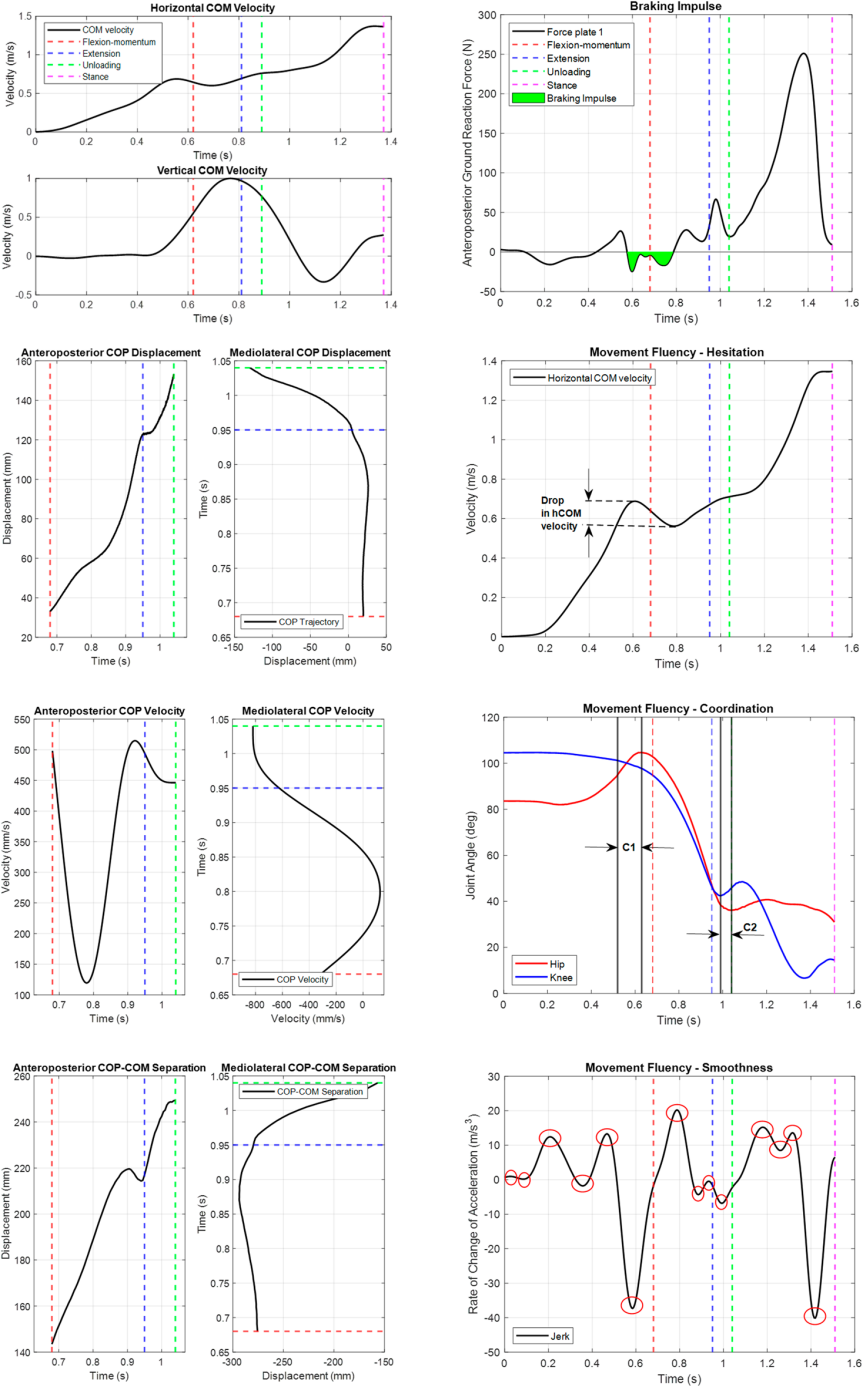
Time series plots of the biomechanical parameters, from a sample subject. The plots include the horizontal centre of mass (COM) and vertical COM velocities, anteroposterior (AP) and mediolateral (ML) center of pressure (COP) trajectories and velocities, AP and ML COP–COM separation, braking impulse and measures of movement fluency—hesitation, coordination (extension occurs at C1 and flexion occurs at C2), and smoothness. The STW events and phases are denoted by vertical dotted lines, where seat-off, gait-initiation (GI), toe-off (TO) of the swing foot and TO of the stance foot, are represented by the end of flexion-momentum (red), extension (blue), unloading (green), and stance (magenta) phases, respectively.

To verify our findings against literature, a series of kinetic, kinematic and movement fluency parameters were derived, based on the four STW transition phases (Fig. 2). These parameters, as illustrated in Fig. 3, were analysed to understand the biomechanics of each of the three classified STW strategies in this study. The derived strategy-wise biomechanics form a generalised framework for STW, which is consistent with the previously established biomechanics for each strategy^2,4,7–9,12^, and can be used to validate the joint angle based strategy classification.

The kinematic and kinetic parameters derived in this study were movement duration, hCOM and vertical COM (vCOM) momentum, braking impulse, COP range and velocities, COP–COM separation and joint angles and torques. Movement duration was obtained from the start of flexion-momentum (beginning of phase 1) to the end of stance (completion of phase 4), which terminates with a TO of the stance foot. Movement initiation was found using the first change in vertical GRF^16^, which also corresponds with the start of an anterior increase in hCOM velocity as the trunk flexes forward^7^. Next, the hCOM velocity at seat-off, represented the hCOM momentum generated during flexion-momentum, and the peak vCOM velocity represented the maximum momentum during extension. Additionally, the posterior GRF during flexion-momentum represented the braking impulse, where the area and peak were considered, as a percentage of bodyweight^7,9^. Moreover, the AP and mediolateral (ML) COP instantaneous velocities and range were obtained as deviations from the force plate centre, while the AP/ML COP–COM separation was the difference between the COM and COP trajectories. Both of these quantities were obtained from extension to unloading phases because, during flexion-momentum there is no significant COP displacement as the feet remain on a single force plate, and during stance, the foot leaves the force plate anteriorly, with gait. Furthermore, the hip/knee joint angles and torques derived from OpenSim were found between seat-off and TO of the stance foot, at points of interest (Table 2). Joint angles were normalised to 0° when the subject was upright and for joint torque, with respect to their bodyweight. The maximum joint torques (not normalised), occurring around seat-off, were also tabulated.

Movement fluency reflects movement mechanical efficiency and comprises three objective measures—hesitation, coordination, and smoothness^11,12^. Hesitation describes an uncertain movement and is the maximum percentage drop in hCOM velocity, from the initial peak before seat-off. Coordination describes the synchroneity of joint movements, via the percentage temporal overlap (with respect to total time) between when the hip/knee move into extension (C1 in Fig. 3) or flexion (C2 in Fig. 3) during the flexion-momentum and unloading phases, respectively. Smoothness describes motion inconsistency and was measured as the total number of inflections in the jerk hCOM signal.

#### Statistical analysis

In this work, SPSS Statistics (IBM) was used to conduct the statistical analysis, with tests using an α of 0.05. This analysis was conducted to find if a statistically significant difference existed between the biomechanical parameters (see the above section), for each STW strategy (Fig. 1); thus, allowing the biomechanics of each strategy to be investigated. The first analysis conducted was to test for normality using the Shapiro–Wilk test, which revealed that the distribution of the dataset was non-parametric. Following this finding, a Kruskal–Wallis H test was selected to find statistical differences between the three STW strategies (independent variable groups) for each biomechanical parameter (dependent variables). Subsequently, for parameters with a statistically significant difference (*P* < 0.05), a Mann–Whitney U test was performed as a post-hoc test, to find how each biomechanical parameter differed between the three strategies, and their extent. Additionally, a power analysis was conducted for the parameters with a statistically significant difference between the strategies, to justify the sample length.

## Results

### Strategy classification

**Figure 4:**
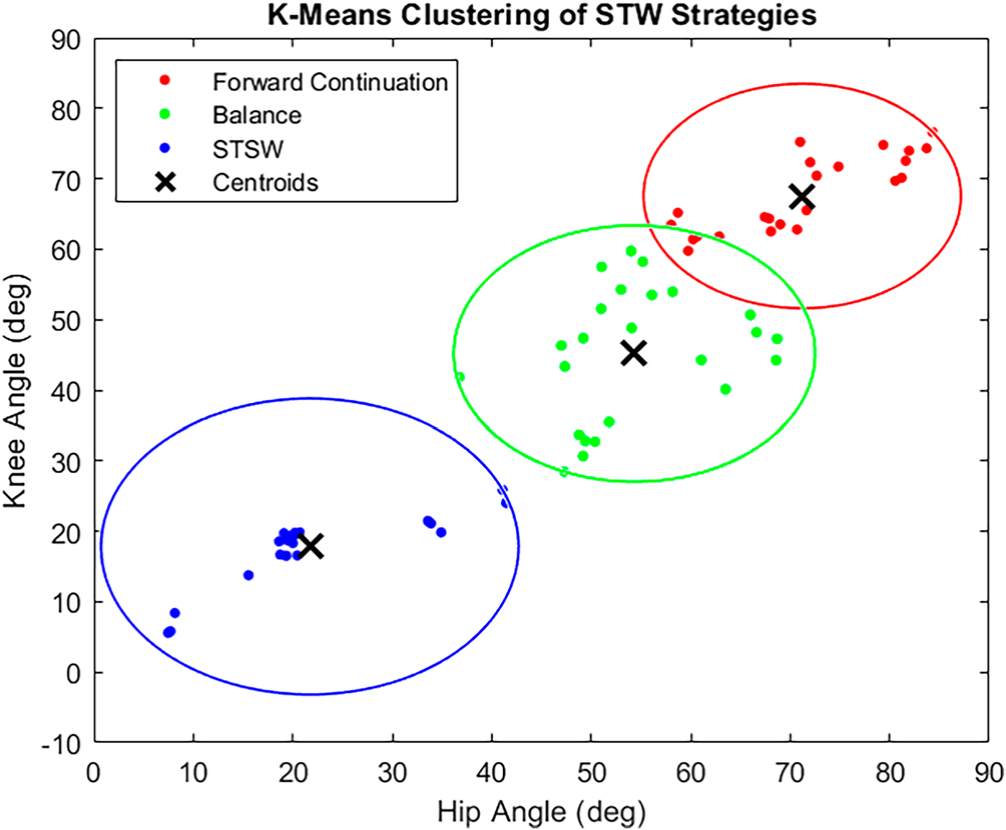
K-means clustering of STW strategies using the hip and knee joint angles at gait-initiation (GI), derived through inverse kinematics from Mocap data. The trend in hip and knee joint angles per strategy is visualized, with forward continuation (red) having the lowest hip and knee extension (largest joint angle magnitude), followed by balance (green) and then STSW (blue), which had the greatest hip and knee extension at GI (smallest joint angle magnitude).

Figure 4 shows the K-means clustering results, which classify the three STW strategies using the hip/knee joint angles at GI, while Table 1 gives their respective joint angle ranges. From this illustration, forward continuation (red) had the lowest hip/knee extension (largest joint angle magnitude), followed by balance (green) and then STSW (blue), which had the highest level of hip/knee extension as the subjects were almost upright at GI. An overlap exists between forward continuation and balance, showing a level of similarity and allowing them to be grouped together as previously reported by Magnan et al.,^7^. Alternatively, STSW acts as a separate postural conservative strategy which is consistent with the findings of Buckley et al.,^2^.

**Table 1:** Hip and knee joint angle ranges for each STW strategy cluster.

Hip and knee joint angle ranges for STW strategy clusters						
Joint angle range (deg)	Forward continuation		Balance		STSW	
	Hip	Knee	Hip	Knee	Hip	Knee
Maximum	84.32	76.63	68.70	59.74	41.45	25.89
Minimum	58.04	59.76	36.75	28.47	7.47	5.58

### Strategy biomechanics

Table 2 presents the statistical analysis of the derived biomechanical parameters, where STW strategy classification through K-mean clustering (Fig. 4), was used when analysing these parameters. From these results, parameters with a statistically significant difference (*P* < 0.05) between the STW strategies (bolded *P*-values in Table 2), were used to describe the variation in biomechanics between each strategy. This is significant, as understanding STW strategy biomechanics in healthy adults would provide reference biomechanical trajectories to aid impaired motion.

**Table 2:** Results of statistical analysis for kinematic, kinetic and movement fluency parameters.

Biomechanical Parameters			(*P* < 0.05) for Kruskal–Wallis H Test	Mann–Whitney U Test (Post-hoc Testing)			Central Tendency and Variability—Median (IQR)		
				Forward & Balance	Forward & STSW	Balance & STSW	Forward	Balance	STSW
Total time (s)			***P* < 0.001**	**0.003**	***P* < 0.001**	**0.003**	1.41 (0.20)	1.68 (0.38)	2.00 (0.13)
Horizontal COM velocity at seat-off (m/s)			***P* < 0.001**	**0.032**	***P* < 0.001**	**0.001**	0.57 (0.09)	0.46 (0.19)	0.35 (0.15)
Peak vertical COM velocity (m/s)			0.893	–	–	–	–	–	–
Braking impulse		Area (Ns)	***P* < 0.001**	**0.005**	***P* < 0.001**	**0.002**	2.40 (2.32)	4.46 (8.82)	15.63 (4.37)
		Peak (N)	**0.025**	**0.028**	**0.015**	0.488	55.28 (30.15)	98.35 (43.83)	86.04 (15.30)
	COP range (mm)	AP	0.748	–	–	–	–	–	–
		ML	***P* < 0.001**	**0.007**	***P* < 0.001**	**0.001**	84.22 (8.22)	113.62 (41.39)	164.14 (48.04)
Instantaneous COP velocity (m/s^2^)	Seat-off	AP	0.343	–	–	–	–	–	–
		ML	0.368	–	–	–	–	–	–
	Gait-initiation	AP	***P* < 0.001**	0.954	***P* < 0.001**	**0.001**	399.94 (124.50)	372.48 (219.33)	− 95.79 (136.69)
		ML	***P* < 0.001**	**0.009**	***P* < 0.001**	**0.011**	− 257.34 (153.97)	− 548.54 (282.38)	− 727.59 (225.19)
	Toe-off swing foot	AP	**0.001**	0.862	***P* < 0.001**	**0.002**	343.01 (208.30)	307.61 (223.16)	− 227.60 (155.65)
		ML	**0.003**	**0.007**	**0.003**	0.273	− 386.48 (230.92)	− 621.44 (246.14)	− 711.57 (263.79)
COP–COM separation (mm)	Seat-off	AP	**0.001**	**0.021**	**0.001**	**0.043**	− 185.25 (46.48)	− 154.54 (21.67)	− 141.21 (18.11)
		ML	**0.010**	0.954	**0.038**	**0.002**	281.79 (27.38)	276.92 (19.06)	264.84 (5.69)
	Gait-initiation	AP	0.111	–	–	–	–	–	–
		ML	0.679	–	–	–	–	–	–
	Toe-off swing foot	AP	0.107	–	–	–	–	–	–
		ML	0.178	–	–	–	–	–	–
Maximum joint torque (Nm)		Hip	0.061	–	–		146.63 (61.77)	133.23 (50.34)	189.32 (47.32)
		Knee	0.152	–	–	–	94.58 (29.31)	84.14 (77.66)	139.10 (59.41)
Normalized joint torque (Nm/kg)	Seat-off	Hip	0.492	–	–	–	–	–	–
		Knee	0.274	–	–	–	–	–	–
	Gait-initiation	Hip	***P* < 0.001**	**0.012**	***P* < 0.001**	***P* < 0.001**	1.68 (0.39)	1.13 (0.66)	0.59 (0.24)
		Knee	***P* < 0.001**	**0.001**	***P* < 0.001**	0.538	0.78 (0.39)	0.29 (0.64)	0.25 (0.19)
	Toe-off swing foot	Hip	***P* < 0.001**	**0.011**	***P* < 0.001**	**0.001**	1.08 (0.43)	0.72 (0.26)	0.42 (0.05)
		Knee	***P* < 0.001**	**0.002**	**0.024**	***P* < 0.001**	1.06 (0.41)	0.70 (0.53)	1.31 (0.17)
	Toe-off stance foot	Hip	**0.007**	**0.003**	0.069	0.094	0.61 (0.20)	0.45 (0.16)	0.52 (0.12)
		Knee	0.558	–	–	–	–	–	–
Movement fluency	Hesitation (%)		***P* < 0.001**	**0.024**	***P* < 0.001**	***P* < 0.001**	13.56 (14.89)	26.34 (34.10)	64.74 (12.01)
	Coordination (%)	C1	***P* < 0.001**	0.386	***P* < 0.001**	**0.001**	− 8.16 (2.03)	− 7.29 (4.04)	− 3.30 (2.42)
		C2	***P* < 0.001**	0.47	***P* < 0.001**	**0.002**	− 3.99 (1.47)	− 3.49 (0.80)	− 2.51 (0.63)
	Jerk		**0.002**	0.155	**0.001**	**0.017**	14.00 (2.50)	15.50 (3.50)	18.00 (2.00)

The bold values indicate statistical significance with (*P* < 0.05).

#### Forward continuation

From Table 2, forward continuation recorded values of shortest duration (median 1.41 s; IQR 0.20), largest hCOM momentum (median 0.57 m/s; IQR 0.09) and lowest braking impulse (median area 2.40 Ns; IQR 2.32) amongst the three strategies. This results in a sharp trunk flexion, to rapidly propel the body forwards and upwards, yet requiring steady balance control to perform without falling. Additionally, the large negative AP COP–COM separation at seat-off (median − 185.25 mm; IQR 46.48) showed the COM lags the COP. This means the feet or BOS is placed further away from the body (Fig. 1), while the large forward momentum keeps balance throughout the motion.

**Figure 5:**
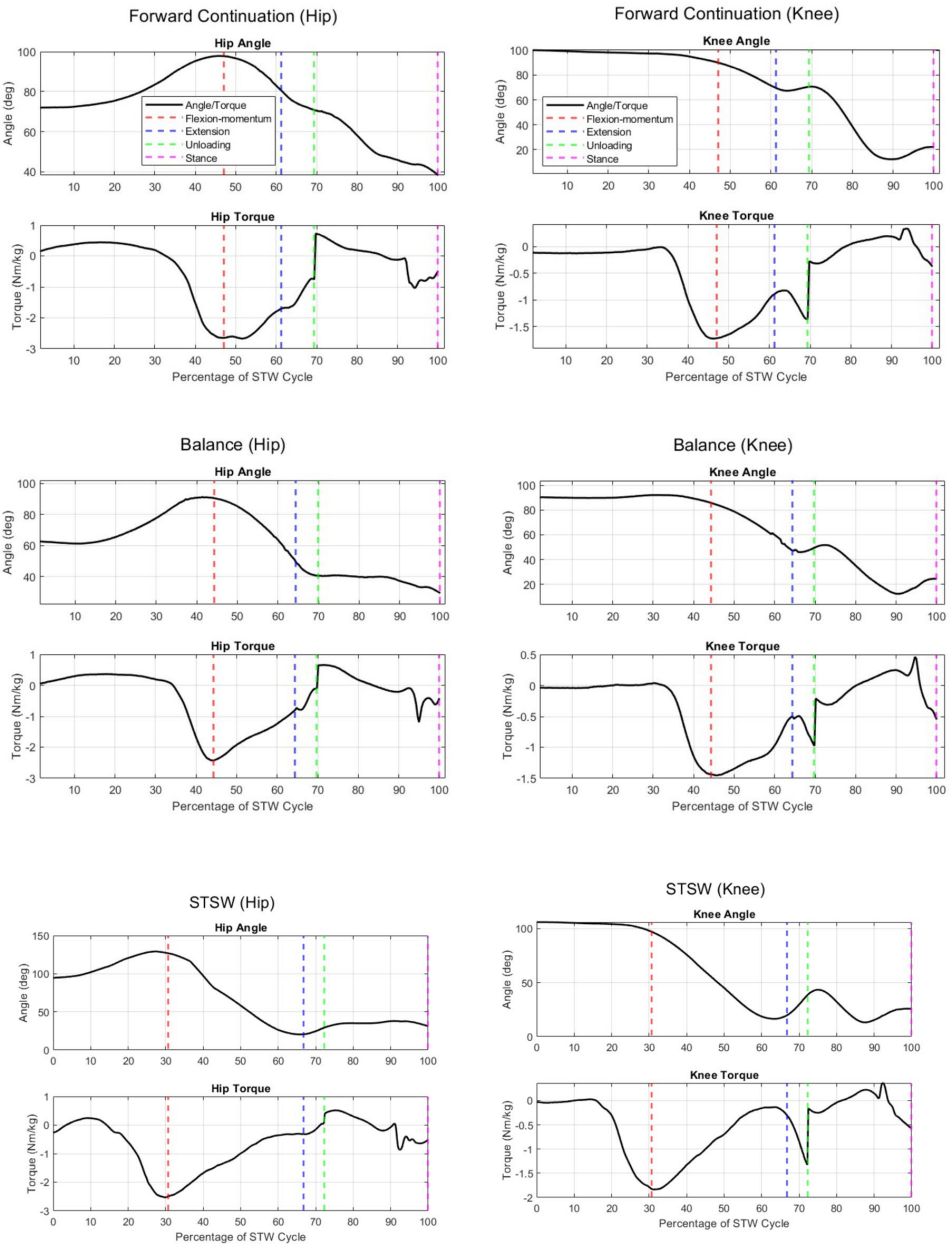
Hip and knee joint angle and torque plots, from selected subjects with respect to the three STW strategies. The STW events and phases are denoted by vertical dotted lines, where seat-off, gait-initiation (GI), toe-off (TO) of the swing foot and TO of the stance foot, are represented by the end of flexion-momentum (red), extension (blue), unloading (green), and stance (magenta) phases, respectively.

Thompson et al.,^30^ showed that COP position and velocities act as balance predictors. Therefore, the lower ML COP range (median 84.22 mm; IQR 8.22) seen in forward continuation could show that this strategy tended to be chosen by individuals with good balance control. From the dataset, this strategy is observed in subjects from 21–40 years and the biomechanics are consistent with the findings of Magnan et al.,^7^ and Rousanoglou et al.,^9^. Moreover, Table 2 shows that forward continuation required greater hip and knee extension (lift) torque to raise the individual. This is due to the lower degree of joint extension as the individual is in a more crouched position when employing this strategy (Fig. 5).

#### Balance

From Table 2, the balance strategy recorded values of longer duration (median 1.68 s; IQR 0.38), lower hCOM momentum (median 0.46 m/s; IQR 0.19) and a higher braking impulse (median area 4.46 Ns; IQR 8.82), compared to forward continuation. The braking impulse reduces forward momentum and allows the individual to focus on stability and postural control, at the cost of speed and efficiency. Additionally, the lower AP COP–COM separation at seat-off (median − 154.54 mm; IQR 21.67), shows the BOS closer to the COM (Fig. 1), allowing the individual to rise while maintaining quasi-static stability.

From this dataset the balance strategy was employed in the 41–50 year age group and in contrast to forward continuation, had greater ML COP range (median 113.62 mm; IQR 41.39) which can indicate a more cautious STW execution^7^. Furthermore, Table 2 recorded lower hip and knee lift torque compared to forward continuation, as the subjects had a greater level of hip and knee extension or were in a less crouched position (Fig. 5).

#### Sit-to-stand-and-walk

STSW reported values of longest duration (median 2.00 s; IQR 0.13), highest braking impulse (median area 15.63 Ns; IQR 4.37) and lowest hCOM momentum (median 0.35 m/s; IQR 0.15), compared to the other strategies (Table 2). The large braking impulse (resulting in lower hCOM momentum) allows the individual to stand while maintaining quasi-static stability and to reach an almost upright position at GI. This is supported by the BOS being closest to the COM as shown by the lowest AP COP–COM separation (median − 141.21 mm; IQR 18.11). Moreover, STSW showed the highest ML COP range (median 164.14 mm; IQR 48.04), velocities, and was observed in the 51–80 year age group, in the current dataset. This may indicate uncertain movements from individuals with less stability and balance control and is consistent with the findings of Buckley et al.,^2^. However, tighter ML COP–COM separation at seat-off (median 264.84 mm; IQR 5.69) was observed, which keeps the feet/BOS in line with the COM, thus preserving movement stability^2,8^. Therefore, STSW allows individuals to maintain stability and balance during ADLs.

Hip and knee torque are progressively lower in STSW (Table 2), as hip/knee extension is greater, and the individual is in a more upright position. As illustrated in Fig. 5, when the joints extend, the required torque decreases from forward continuation to balance to STSW. However, during TO of the swing foot, the knee flexes, as gait begins. Due to the larger knee extension in STSW, the knee must move through a greater range of motion and higher knee flexion torque is required at this point.

Finally, movement fluency significantly differed (*P* < 0.05) between STSW with the other strategies (Table 2), as similarly observed by Jones et al.^10^. For STSW, a large braking impulse results in a greater hCOM momentum drop—greater hesitation and lower smoothness^11,12^. Moreover, negative values and smaller magnitudes show weaker coordination as the knee moves into extension/flexion just before the hip. This further emphasises that STSW is prone to be employed when an individual is uncertain of their motion, with weak motor control, balance, and stability.

## Discussion

Strategy classification using the hip and knee joint angles at GI is important, as it provides a framework for identifying STW biomechanics^2,7–10,12^, based on three generalised strategies. While individuals would have varied STW biomechanics, the strategies capture the general biomechanical differences between STW executions. This would inform the design of assistive devices, as biomechanical trajectories of healthy adults (for example, strategy-specific joint torque) would act as a reference/benchmark for assisting impaired motion^17,18^. Tailored assistance can also be provided by considering the varying strategy joint torque requirements, rather than generic STW assistance. Moreover, the study had ten subjects and used SMOTE to equalise sample lengths, with twelve trials per strategy in total. Based on this, the power analysis results (for a significance of *P* < 0.05), showed that the average statistical power for the biomechanical parameters that significantly varied with strategy, was 0.94 (SD = 0.14). This shows a 6% probability of a Type II error and sufficient statistical power for the results of this study to be considered significant.

K-means clustering was performed for STW strategy identification using the hip/knee joint angles, and these findings were used to analyse the variation in biomechanics (derived in this study) between each STW strategy (Table 2). The observed variation in STW execution biomechanics (for example, hCOM momentum or braking impulse) are consistent with the established biomechanics in literature—describing the three STW strategies^2,7–10,12^. This demonstrates correct application of the K-means cluster grouping and thus, validates the use of hip/knee joint angles at GI, as an alternate method of STW strategy classification. In literature, complete STW motion biomechanics were considered to reliably distinguish the STW strategy. However, joint angles are beneficial as they serve as a standalone parameter and are obtained during motion. The advantage of joint angles is that they can be measured through wearable sensors with reduced post-processing; which allows integration with real-time applications such as assistive devices and minimizes the need for expensive laboratory setups^14,15^.

A typical individual (regardless of age) can employ either strategy at different instances, where the central nervous system selects the best strategy. Therefore, all three STW strategies and biomechanics should be considered when designing assistive devices, as they must support an individual to perform their chosen strategy, in a natural manner. Biomechanical trajectories of healthy adults, like hip/knee joint torque (Fig. 5), would act as a reference for assisting impaired motion^17,18^, while providing strategy-specific and user-targeted assistance. This study categorised the three strategies but did not reproduce the subject kinematics/kinetics per strategy. Yet, strategy classification could inform the evaluation process of assistive devices. For a device to be representative of STW, it should provide correct/sufficient assistance regardless of the employed strategy. Only if it can perform for all three STW strategies, would it encompass the entire STW motion—describing its efficacy, effectiveness, and applicability to STW.

On that account, lift assistive devices should consider the different strategy-wise torque variations, to ensure correct levels of assistive torque are provided. Forward continuation required the largest extension torque, followed by balance. In contrast, STSW required less extension torque, but greater knee flexion torque at TO. This result, coupled with lower hCOM momentum, tighter AP and ML COP–COM separation, and a larger braking impulse, showed that STSW resulted in better stability during ADLs. Therefore, older adults are more prone to employing STSW^2^, while balance and forward continuation strategies require better postural and balance control^7^. This is observed in the dataset, where subjects between 51–80 years tended to use STSW while middle aged and younger adults were prone towards forward continuation or balance.

A limitation is that this study only considered torque at points of interest and did not investigate the overall STW torque profile, per strategy. This is because the focus was on analysing STW motion biomechanics, based on the transition phases (Fig. 2), to identify strategy specific differences for classification. Furthermore, the study did not investigate upper limb motion such as arm strategies^4^, asymmetric foot position or the use of walking aids as commonly used during ADLs. Upper body dynamics would have a reduced effect on lower body movement biomechanics^2,7–9^ but would affect parameters such as momentum generated or balance control and should be researched in future studies.

## Conclusion

This study proposed using the hip/knee joint angles at GI with K-means clustering, as an alternate method of classifying the three STW strategies—forward continuation, balance and STSW. An individual can employ either strategy at an instance; yet, with ageing, as movement patterns change, older adults would be prone to using STSW, while middle and younger adults would be prone towards balance and forward continuation. Joint angle strategy classification is useful as it can be measured with wearable sensors and integrated into real-time applications. To validate the strategy identification method, the strategy-wise biomechanics were derived, based on the joint angle strategy classification, Mocap, and GRF data; and was found to be consistent with existing literature. The biomechanical parameters governing STW strategies are hCOM momentum, braking impulse, ML COP range, COP–COM separation at seat-off, joint torque and movement fluency. These biomechanics form a generalized framework for describing STW executions in healthy adults and would aid the design and evaluation of assistive devices, by acting as a reference to assist impaired motion, thereby improving access to ADLs.

### Supplementary Information

Supplementary Information.

## Data Availability

All datasets analyed during this study are included in the published article by Liang et al.,^20^ and can be found under the following hyperlink: 10.1038/s41597-020-00627-7

## Abbreviations

ADL: Activity of daily living
AP: Anteroposterior
COM: Centre of mass
COP: Centre of pressure
GI: Gait initiation
GRF: Ground reaction force
hCOM: Horizontal centre of mass
IQR: Interquartile range
ML: Mediolateral
Mocap: Motion capture
SMOTE: Synthetic minority oversampling technique
STS: Sit-to-stand
STSW: Sit-to-stand-and-walk
STW: Sit-to-walk
TO: Toe-off
TUG: Timed-up-and-go
vCOM: Vertical centre of mass
