# Supplementary material for: Sit-to-walk strategy classification in healthy adults using hip and knee joint angles at gait initiation
Source: Sci Rep. 2023 Oct 3;13:16640. doi: 10.1038/s41598-023-43148-0 (PMC10547676; doi:10.1038/s41598-023-43148-0)
Supplement: Supplementary file 1 — Supplementary Information. [file 41598_2023_43148_MOESM1_ESM.pdf]

Supplementary Information

OpenSim Procedure

Manuscript title: Sit-to-walk strategy classification using hip and knee joint angles at gait initiation

Authors names

Chamalka Kenneth Perera[1], Alpha Agape Gopalai[1*], Darwin Gouwanda[1], Siti Anom Ahmad[2], Mazatulfazura Sf Binti Salim[2]

[1] School of Engineering, Monash University, Selangor, Malaysia

[2]Malaysian Research Institute on Ageing, Universiti Putra Malaysia, Selangor, Malaysia

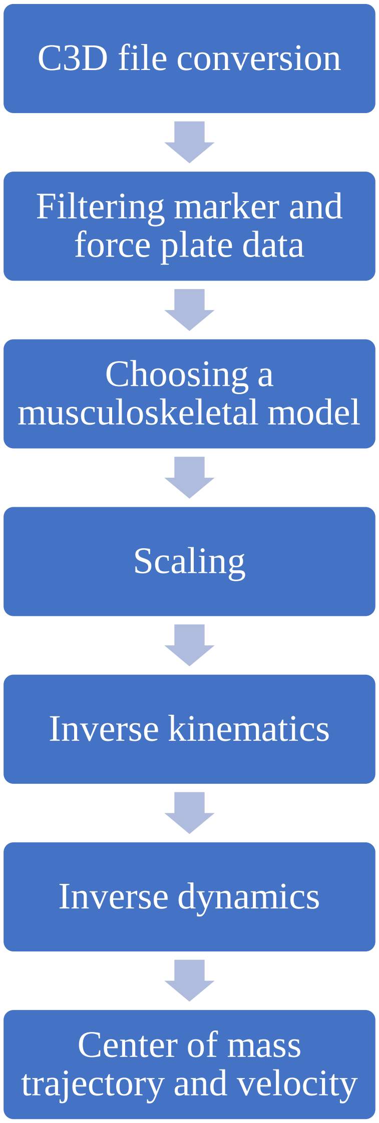

Figure 1. OpenSim data processing procedure.

Figure 1 shows an outline of the OpenSim (version 4.2)[1,2] data processing procedure. The raw subject motion capture (Mocap) and force plate data, were obtained from an open access dataset, performed by the Rehabilitation Research Institute of Singapore[3], available through the NTU Dataverse database in C3D format.

C3D file conversion

The C3D files from the dataset were first converted into '.trc' and '.mot' formats as required by OpenSim, for Mocap marker and ground reaction force (GRF) data, respectively. The file conversion was performed using the MATLAB-OpenSim API. During this process, the global coordinate system is rotated, such that the X-axis points in the anteroposterior direction, the Z-axis is in the mediolateral direction and the Y-axis points vertically upwards (Figure 2). The columns in the GRF data file (.mot) were re-ordered so that the GRF vector columns (Vx, Vy and Vz) preceded the ground reaction centre of pressure (COP) columns (Px, Py, Pz) for all force plates, in order. This was then followed by the ground reaction moment vector columns (torque_x, torque_y, torque_z) in order of the force plates.

Filtering

The raw Mocap marker and GRF data contained noise, such as sensor (white) noise, marker wobbling noise, motion artifacts and quantization noise – hence, were filtered prior to analysis[4]. The marker and GRF sampling frequencies were 200Hz and 2000Hz, respectively. The filter cut-off frequencies were determined by observing the 99% occupied bandwidth of the signal frequency domain via a Fast Fourier Transform. The raw data was filtered using a zero-lag second order Butterworth low pass filter (LPF) at cut-off frequencies of 5 Hz for Mocap data and 20 Hz for GRF data[5–7]. The cut-off frequency for GRF data was selected to preserve motion events at the point of gait-initiation.

Gait2392 musculoskeletal model

The Gait2392 musculoskeletal model (provided by OpenSim) was chosen for this study (Figure 2). The model is primarily a lower body model with two legs and a torso segment, making it suitable for analysing the hip and knee movements during sit-to-walk (STW)[8]. The coordinates, names, and number of generic model markers were edited to match the subject data, from the open access dataset[3]. The modified marker set was saved ('in .xml' format) for use in scaling. All '.xml' files used, were obtained from the OpenSim sample files[9] and were modified accordingly.

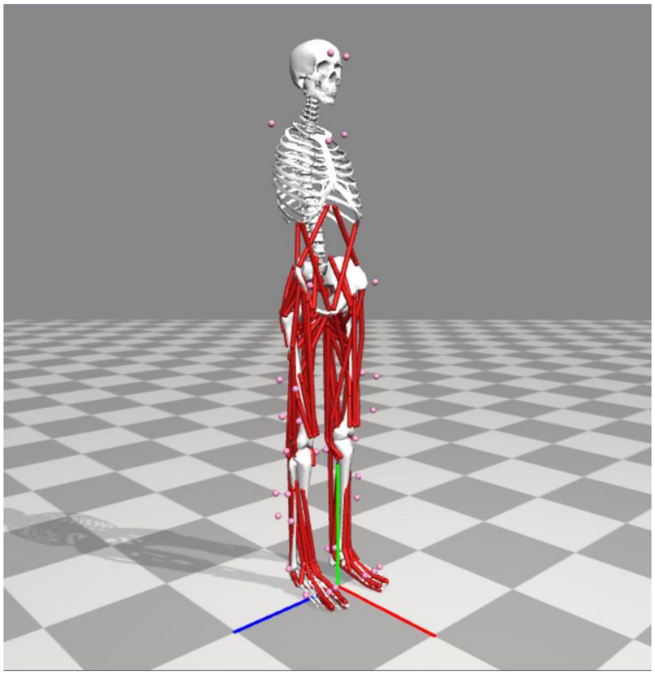

Figure 2. Gait2393 generic OpenSim musculoskeletal model with model markers (pink) to match the subject's experimental marker positions. The X-axis (red) is in the anteroposterior direction, while the Z-axis (blue) is in the mediolateral direction.

Scaling

Scaling is the process of matching the generic musculoskeletal model to suit the anthropometry of a subject. Measurement-based scaling was performed, using the Mocap markers placed on bone landmarks. The inputs to the scale tool were the marker set and the Mocap static trial in '.trc' format. A scale setup file was created ('.xml' file format) with pointers to the input files and definitions for each body segment based on the distance between the marker pairs placed on the relevant landmarks[10]. For example, the thigh segment was created using the ASIS (anterior superior iliac spine) marker and the FLE (femur lateral epicondyle) marker, for both left and right sides. Similarly, the torso, pelvis, shank, and foot segments were also created. Based on this, scale factors were assigned to the relevant bone segments.

Additionally, static pose weights were assigned for each marker. This weightage affects the degree to which the virtual and experimental markers can deviate, where a higher weightage would allow for a lower marker error. Segment definition markers on bone landmarks (head, sternum, knee and ankle joints) had a higher weightage of 1000.0, while tracking markers had a lower weightage of 1.0 [9].

Scaling is an iterative process, which continues until the root mean square (RMS) and maximum marker errors are less than 1 cm and 2 cm, respectively. During this process, the scaled model is previewed with each iteration, to observe the marker error (messages window). The 3D coordinates of the virtual model markers are adjusted until they closely match the experimental marker coordinates[10]. Once the errors were within the recommended limits, the scale tool was re-run (without preview), and the virtual and experimental markers were allowed to coincide. The output of scaling was a subject specific scaled musculoskeletal model (Figure 3).

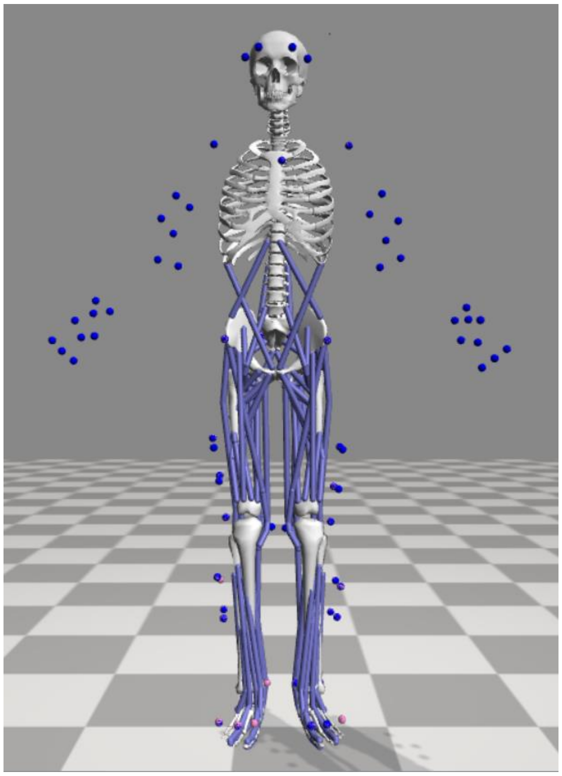

Figure 3. Subject specific scaled musculoskeletal model with coinciding experimental (blue) and virtual (pink) markers.

The scaled model was verified by first observing the hip, knee, and ankle joint angles in the static position. For example, hip angle while standing should be less than ten degrees flexion, ankle angle should be approximately five degrees and knee angle should be approximately zero degrees[10]. Furthermore, the centre of mass (COM) and line of action of the vertical GRF were observed during sit-to-walk (STW). The position of the COM during sitting, was as described in literature[11], and the vertical GRF matched the COM trajectory in the anteroposterior and mediolateral directions, during standing, as illustrated in Figure 4. This shows a good fit between the GRF (experimental) data and the model's movements.

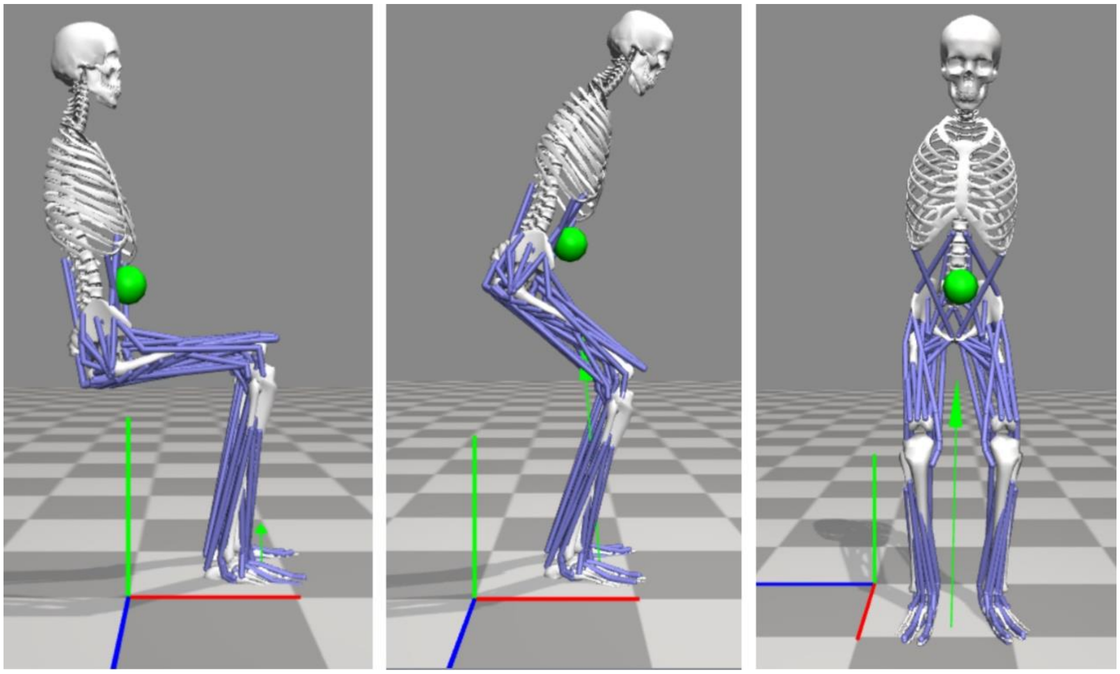

Figure 4. Validation of the scaled model using the COM (green ball) and the vertical GRF (green vertical arrow) when standing.

Inverse kinematics

The dynamic trial data was assigned to the scaled model and inverse kinematics (IK) was computed (via the IK tool), to find the generalized coordinate values (joint angles), as illustrated in Figure 5. The inputs to the IK tool were the dynamic Mocap data (in '.trc' format) and marker weights. Marker weightage and pointers to input files were stored in an IK setup file of '.xml' format. IK runs (using a sum of weighted least square errors of markers model) through each time frame of the Mocap data and positions the scaled model in a pose that best matches the experimental marker coordinates[9].

The weightage applied to each marker is relative, where a larger weight means tighter tracking of the virtual to the experimental markers. A larger weight produces less marker error and is assigned to markers that experience less deviation. Segment definition markers on bone landmarks were assigned a weight of 10.0, while tracking markers were assigned a weight of 1.0. The output from IK was a '.mot' file, containing all the relevant joint angles[9].

To verify the IK solution, the joint angles for the hip, knee and ankle were plotted (during gait) and compared with existing plots from literature. The hip and ankle joint angle plots matched plots in existing literature[12], yet, the knee angle plot was inverted. This is due to the negative sign convention for knee flexion, used by OpenSim.

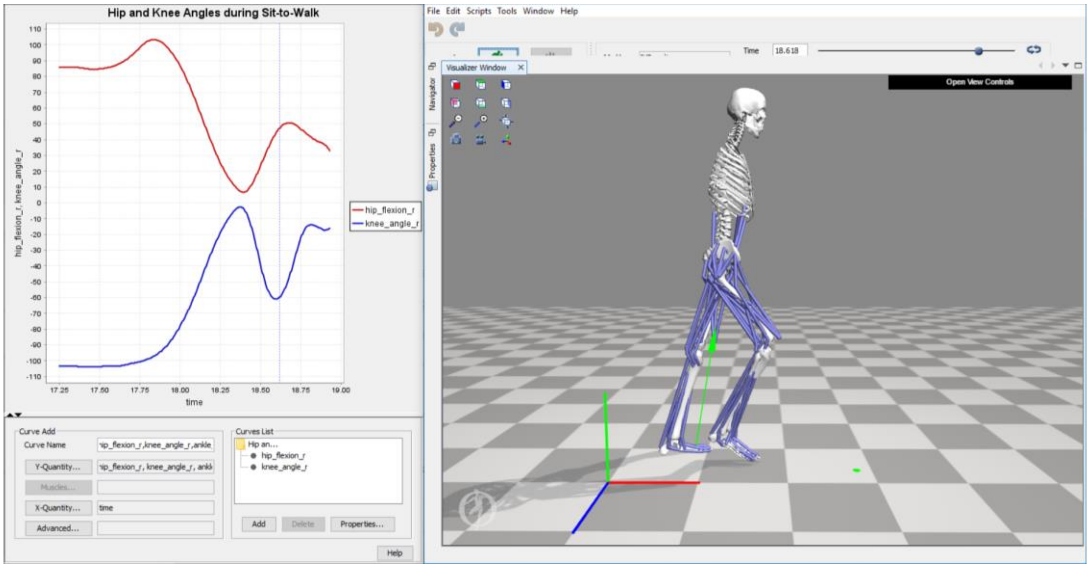

Figure 5. The scaled musculoskeletal model during the sit-to-walk movement, after the IK solution was computed. The plot shows the hip (red) and knee (blue) joint angles for the right foot during sit-to-walk.

Inverse dynamics

Following the IK solution, inverse dynamics (ID) was computed, to calculate the net joint forces and moments (joint torque). The inputs to the ID tool consisted of the IK solution and GRFs from the force plates, which were specified in an ID setup file of '.xml' format. ID was calculated using the Newton-Euler equations of motion[9].

A 6Hz LPF was applied within OpenSim for the data from the IK solution. This was to reduce the noise produced in the second order derivatives, which stem from the relevant equations of motion[13]. The GRF from each force plate was assigned as a point force acting on the Calcaneus (calc) of each foot, expressed in the ground plane, created under the External Loads tab, and saved in '.xml' format. As the subject moves from one force plate to another, the GRF acting on the swing foot changes between the force plates. Therefore, two ID solutions were computed, as the point force acting on the swing foot changed from the first to the second force plate, after toe-off. The two ID results were then concatenated to form a single dataset. It should be noted that only the joint torques within the force plate regions are valid. Once the subject leaves the force plates,

OpenSim would assume only gravity acts on the subject, resulting in an incorrect estimation of the joint torque.

The joint torque can be inconsistent due to marker noise, motion artifacts, and modelling inaccuracies. Noise may be introduced due to the first and second order differentiation of experimental marker coordinate data, to joint velocities and accelerations, respectively. Modelling errors are primarily due to inconsistencies in the musculoskeletal model geometry and mass distribution. A lumped torso segment is used in the Gait2392 model, which does not account for neck and head movement. It also restricts complex trunk bending movement due to the flat nature of model's spinal segment, which can lead to errors in the model geometry. Furthermore, as no arms are present in the model, the mass and inertial properties may also lead to modelling inaccuracies. These experimental errors and modelling inconsistencies may affect how the equations of motion are computed in ID[8,14].

Residuals are non-physical compensatory forces that form due to the inconsistencies in GRF data and between experimental and model marker data[9,15]. The presence of such residuals are beneficial (should not be reduced to zero), as they may account for modelling assumptions, such as the lack of arms and a lumped torso[16]. OpenSim runs a residual reduction algorithm (RRA) which is intended for gait (walking and running) and minimizes the residuals but does not remove them entirely[9]. Due to the sit-to-walk movement in this study, when RRA was computed, the increase in vertical GRF while the subject stands (both feet on a single force plate) before seat-off, is interpreted incorrectly, causing the musculoskeletal model to move vertically downwards (through the ground plane) and then upwards (above the ground plane). Hence, as sit-to-walk is considered a single fluid movement, which includes sit-to-stand and gait initiation, RRA was not performed on the subject data. Instead, the ID solution was used to obtain the joint torques, given an acceptable residual[9,17].

COM trajectory and velocity

OpenSim was also used to calculate the COM trajectory and velocity. This was performed using the Analyse tool with a BodyKinematics analysis. The input was the IK solution and a LPF with a cut-off frequency of 6Hz was applied (within OpenSim), prior to calculating the COM kinematics.

Like ID, two sets of COM kinematics were obtained due to different force plate definitions caused by the swing foot moving from one force plate to the other. This was specified under the Actuators and External Loads tab, while the two solutions were concatenated to form a single dataset.

References

1. Delp, S. L. *et al.* OpenSim: open-source software to create and analyze dynamic simulations of movement. *IEEE Trans Biomed Eng* **54**, 1940–1950 (2007).

2. Seth, A. *et al.* OpenSim: Simulating musculoskeletal dynamics and neuromuscular control to study human and animal movement. *PLOS Computational Biology* **14**, e1006223 (2018).

3. Liang, P. *et al.* An Asian-centric human movement database capturing activities of daily living. *Sci Data* **7**, (2020).

4. Skogstad, S., Nymoen, K., Høvin, M., Holm, S. & Jensenius, A. Filtering Motion Capture Data for Real-Time Applications. in (NIME, 2013).

5. Koller, J. R., Remy, C. D. & Ferris, D. P. Biomechanics and energetics of walking in powered ankle exoskeletons using myoelectric control versus mechanically intrinsic control. *Journal of NeuroEngineering and Rehabilitation* **15**, 42 (2018).

6. Sinclair, J., Taylor, P. J. & Hobbs, S. J. Digital Filtering of Three-Dimensional Lower Extremity Kinematics: an Assessment. *J Hum Kinet* **39**, 25–36 (2013).

7. Yu, B., Gabriel, D., Noble, L. & An, K. N. Estimate of the optimum cutoff frequency for the Butterworth low-pass digital filter. *Journal of Applied Biomechanics* **15**, 318–329 (1999).

8. OpenSim. Musculoskeletal Models - OpenSim Documentation. https://simtk-confluence.stanford.edu/display/OpenSim/Musculoskeletal+Models (2021).

9. OpenSim. User's Guide - OpenSim Documentation. https://simtk-confluence.stanford.edu/display/OpenSim/User%27s+Guide (2021).

10. OpenSim. Scaling Best Practices - OpenSim Documentation. https://simtk-confluence.stanford.edu/display/OpenSim/_Scaling+Best+Practices (2021).

11. Cacciatore, T. & Johnson, P. The Physics of Sit-to-Stand – Alexander Technique Science. https://www.alexandertechniquescience.com/biomechanics/the-physics-of-sit-to-stand/ (2021).

12. Aucie, Y., Zhang, X., Sargent, R. & Torres-Oviedo, G. Motorized Shoes Induce Robust Sensorimotor Adaptation in Walking. *Front. Neurosci.* **0**, (2020).

13. van den Bogert, A. & de Koning, J. ON OPTIMAL FILTERING FOR INVERSE DYNAMICS ANALYSIS. (1996).

14. Roelker, S. A. *et al.* Interpreting Musculoskeletal Models and Dynamic Simulations: Causes and Effects of Differences Between Models. *Ann Biomed Eng* **45**, 2635–2647 (2017).

15. Samaan, M. A., Weinhandl, J. T., Bawab, S. Y. & Ringleb, S. I. Determining residual reduction algorithm kinematic tracking weights for a sidestep cut via numerical optimization. *Comput Methods Biomech Biomed Engin* **19**, 1721–1729 (2016).

16. Anderson, F., John, C., Guendelman, E., Arnold, A. & Delp, S. SimTrack : Software for Rapidly Generating Muscle-Actuated Simulations of Long-Duration Movement. https://www.semanticscholar.org/paper/SimTrack-%3A-Software-for-Rapidly-Generating-of-Anderson-John/653e1f55a41a3740494884c9184a7bedfe1ba47f (2006).

17. Hamner, S. R., John, T., Anderson, F. C., Higginson, J. S. & Delp, S. L. Reducing Residual Forces and Moments in a Three-Dimensional Simulation of Running. https://www.academia.edu/3729095/REDUCING_RESIDUAL_FORCES_AND_MOMENTS_IN_A_THREE_DIMENSIONAL_SIMULATION_OF_RUNNING (2021).
